# Supplementary material for: Antiproliferative Activity of Krukovine by Regulating Transmembrane Protein 139 (TMEM139) in Oxaliplatin-Resistant Pancreatic Cancer Cells
Source: Cancers (Basel). 2023 May 7;15(9):2642. doi: 10.3390/cancers15092642 (PMC10177337; doi:10.3390/cancers15092642)
Supplement: Supplementary file 1 [file cancers-15-02642-s001.zip › cancers-2299694-supplementary.pdf]

Supplementary Figure S1

a

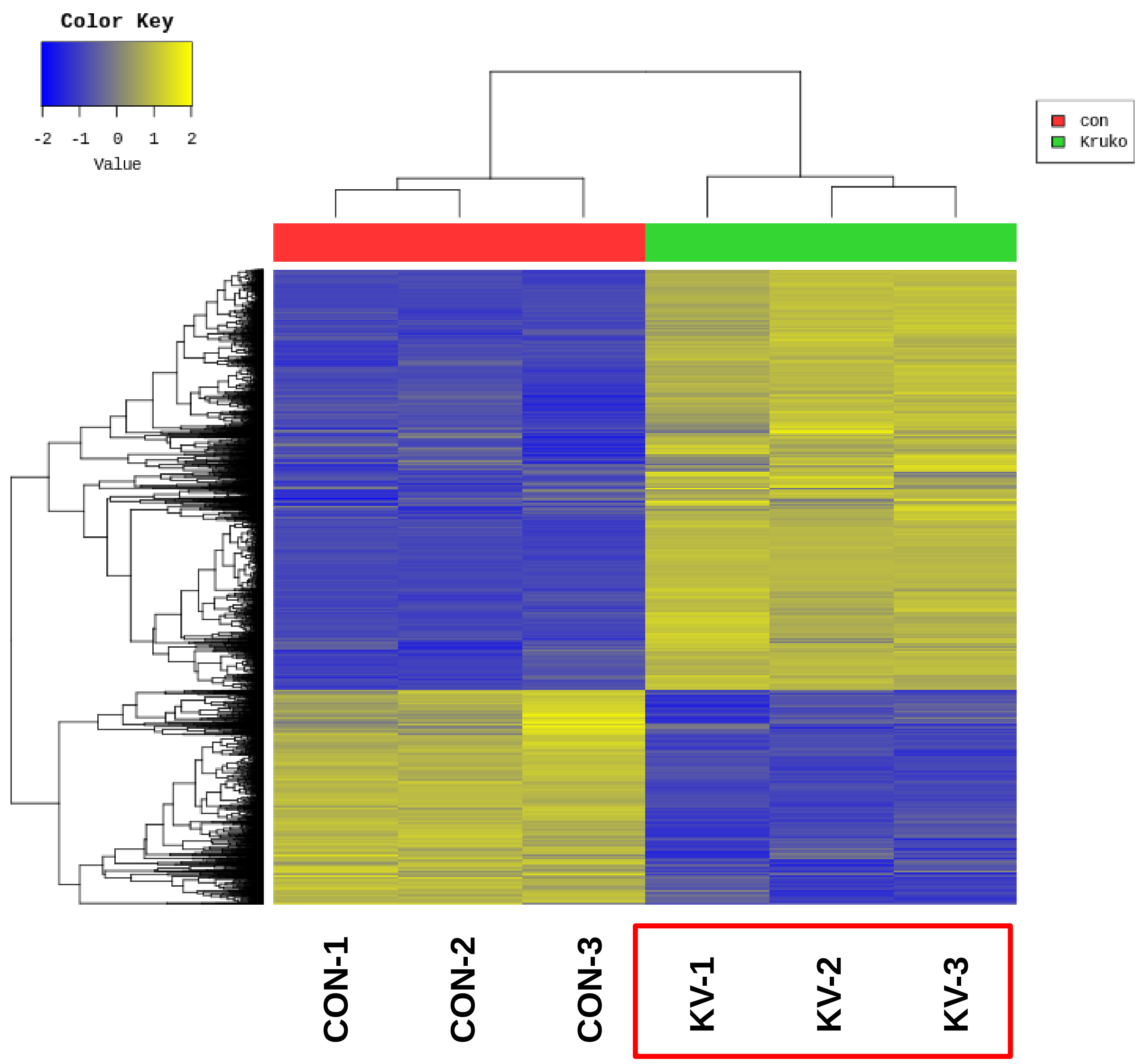

RNA-sequencing data (oxaliplatin-resistant AsPC-1 cells were treated with KV (25 μM) for 48 h. Heatmap of one-way hierarchical clustering using z-score ($-2 \leq z \leq 2$) for normalized value (log2 based) (4,269 genes satisfying with fc 2 & raw.p)
All data represent at least three independent experiments.

b

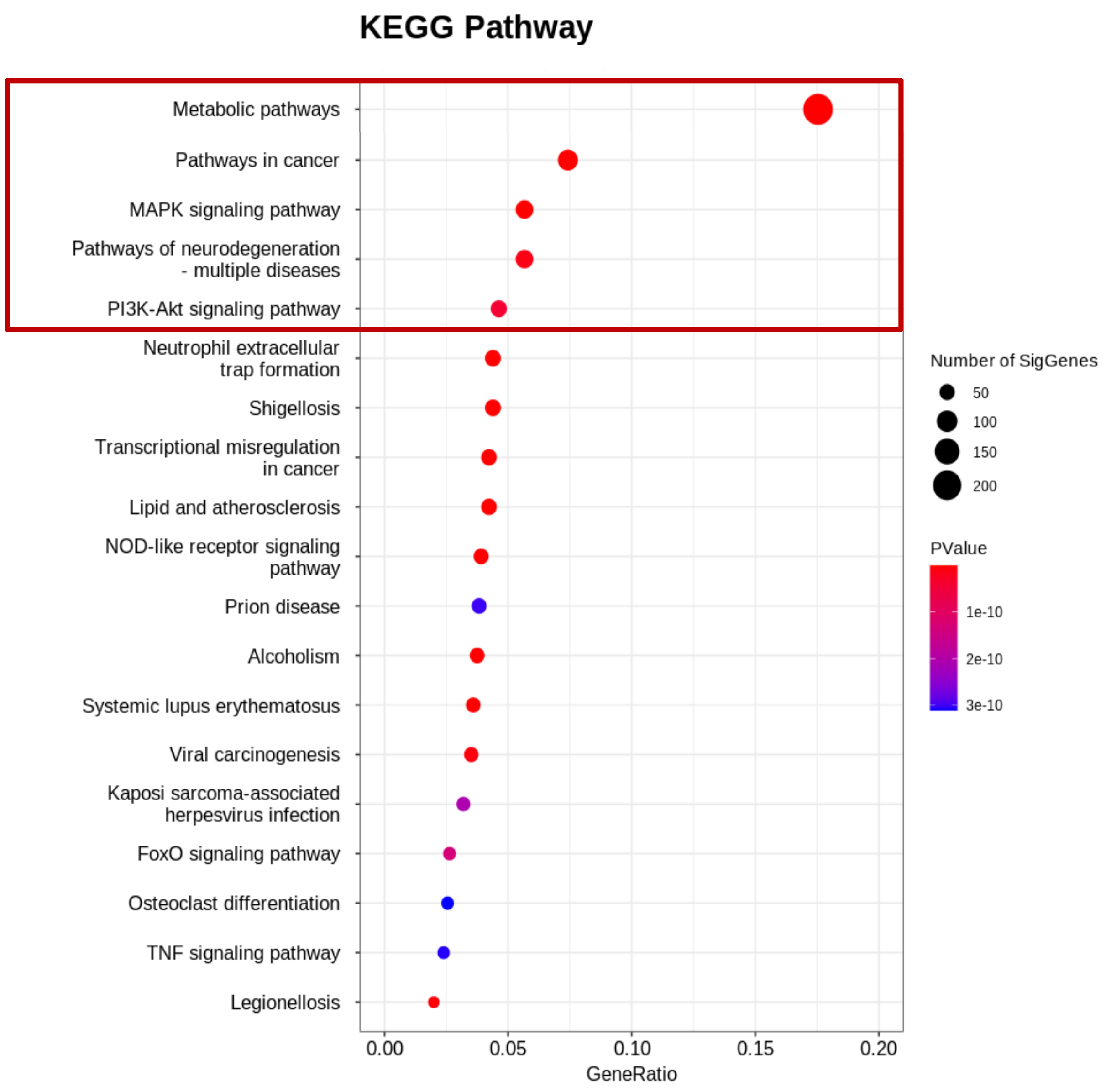

Top 19 terms of KEGG pathway. The dot color represents the p-values. The scale of the spots indicates the number of genes involved.

Supplementary Figure S2

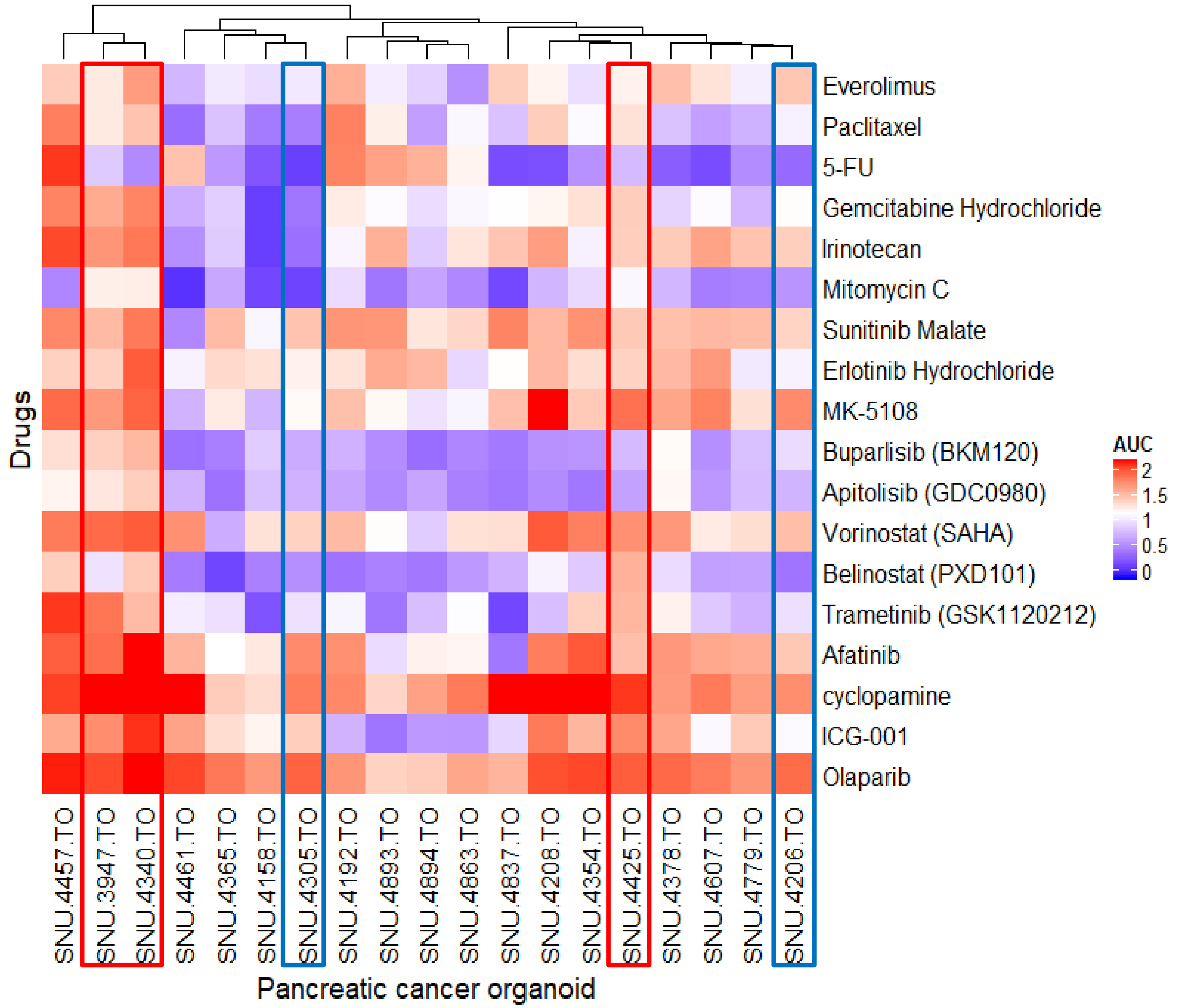

Multi-drug response heatmap, multi-drug response on PDPCOs
