# Supplementary figures and images for: Antiproliferative Activity of Krukovine by Regulating Transmembrane Protein 139 (TMEM139) in Oxaliplatin-Resistant Pancreatic Cancer Cells
Source: Cancers (Basel). 2023 May 7;15(9):2642. doi: 10.3390/cancers15092642 (PMC10177337; doi:10.3390/cancers15092642)

## Slide 1
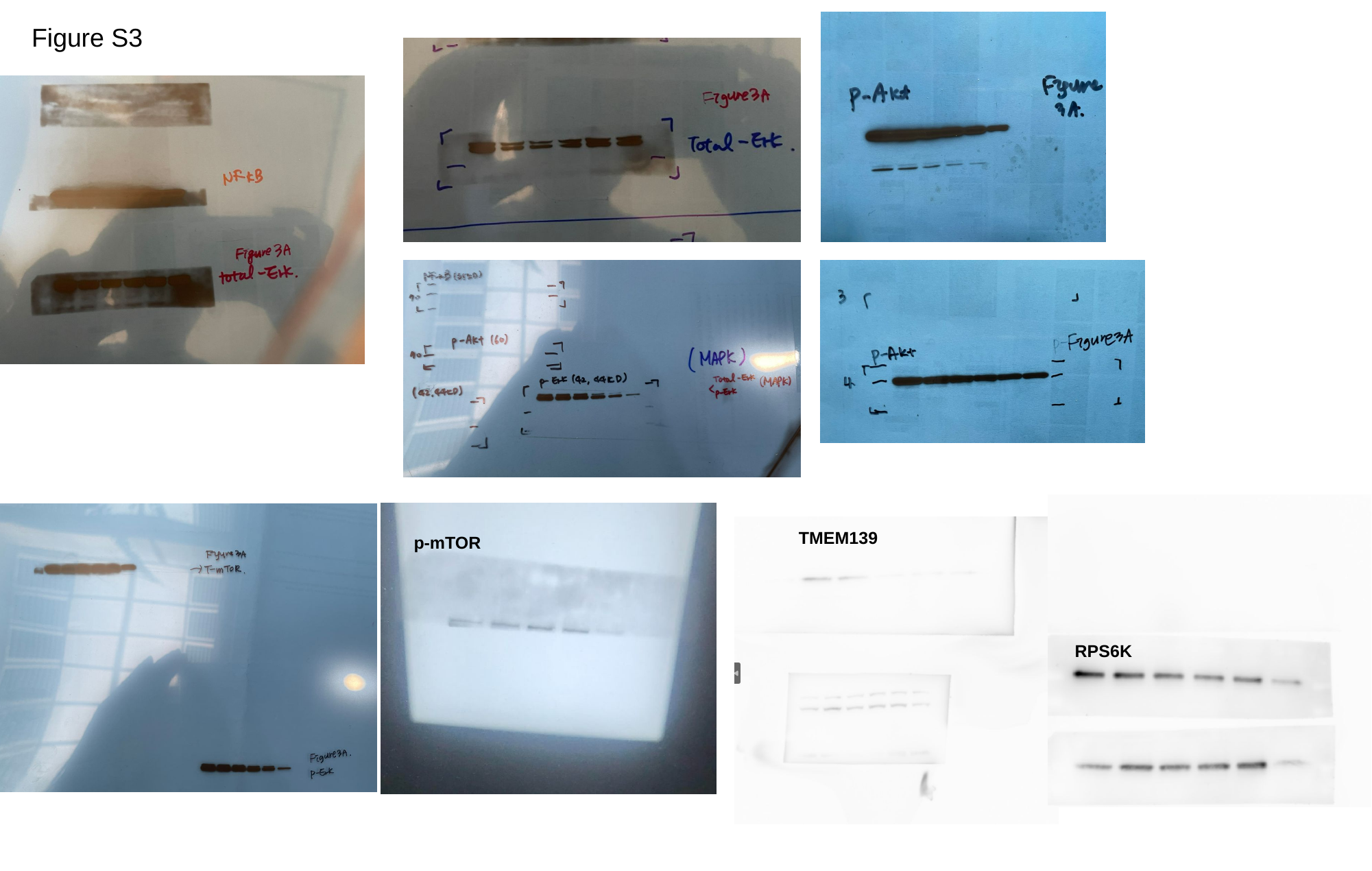

Figure S3
TMEM139
p-mTOR
RPS6K

## Slide 2
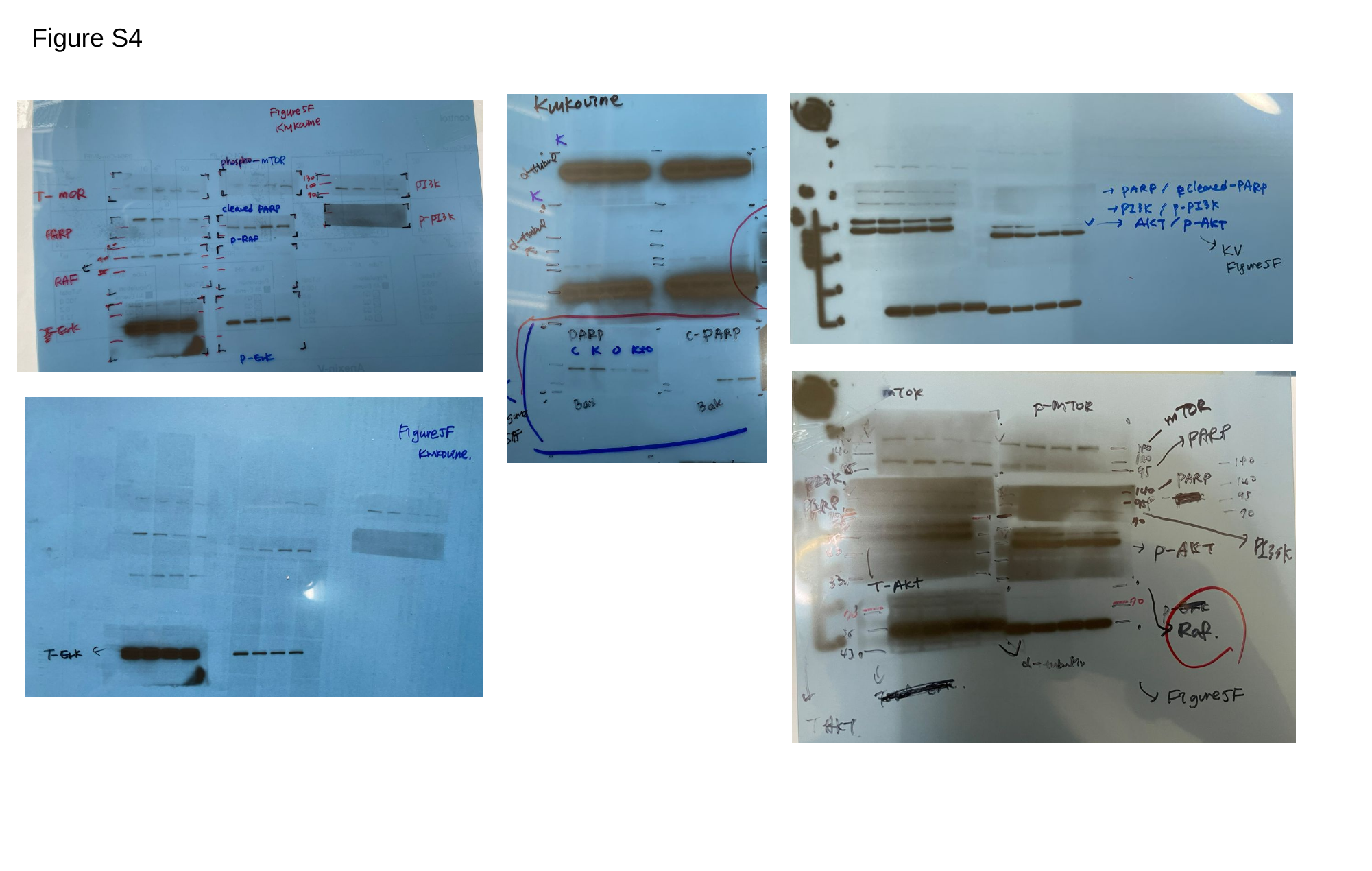

Figure S4

Supplement: Supplementary file 1 [file cancers-15-02642-s001.zip › Krukovine_Supplementary data_23.05.06 (Figure S3, Figure S4).pptx]
